# Absence of uterus and presence of verumontanum in a 46 XX patient with Congenital adrenal hyperplasia reared as male: A case report with literature review

**DOI:** 10.1016/j.eucr.2025.103028

**Published:** 2025-03-26

**Authors:** Rawa Bapir, Ismaeel Aghaways, Shaho F. Ahmed, Nali H. Hama, Ari M. Abdullah, Soran H. Tahir, Zana B. Najmadden, Fahmi H. Kakamad, Aso N. Qadir, Tomas M. Mikael

**Affiliations:** a Smart Health Tower, Madam Mitterrand Street, Sulaimani, Kurdistan, Zip code: 46001, Iraq; b Kscien Organization, Hamdi Street., Azadi Mall, Sulaimani, Kurdistan, Zip code: 46001, Iraq; c Department of Urology, Surgical Teaching Hospital, Sulaimani, Kurdistan, Zip code: 46001, Iraq; d College of Medicine, University of Sulaimani, Madam Mitterrand Street, Sulaimani, Kurdistan, Zip code: 46001, Iraq; e Research Center, University of Halabja, 46018, Halabja, Kurdistan Region, Zip code: 46001, Iraq

**Keywords:** Ambiguous genitalia, 21-Hydroxylase deficiency, Verumontanum

## Abstract

Congenital adrenal hyperplasia (CAH) is an inherited disorder causing adrenal hormone imbalance and organ overgrowth, leading to phenotype-genotype mismatches. A 10-year-old phenotypic male with impalpable testes and hypospadias had ambiguous genitalia and an empty scrotum. Imaging showed intra-abdominal gonads and no uterus. Blood tests revealed low cortisol, high ACTH, and 17-OHP. Karyotyping confirmed 46 XX. CAH can virilize 46 XX individuals, sometimes resulting in male assignment. Late presentation with male identity may support gender choice for better psychological outcomes.

## 1 Background

Congenital adrenal hyperplasia (CAH) is a family of autosomal recessive disorders that results in a deficiency of enzymes needed for the synthesis of cortisol in the adrenal cortex. The most common cause of CAH is the deficiency of the enzyme 21-hydroxylase.^1^ The incidence of CAH varies among different ethnic and racial groups. Generally, the incidence of the classic form is 1 in 16,000 births. The incidence of Non-Classical Congenital Adrenal Hyperplasia (NCAH) is higher than that of the classic form, occurring at a rate of 1 in 1000. The prevalence of NCAH is more common in specific populations like Hispanics, Yugoslavs, and Ashkenazi Jews.^1^^,^^2^

Here, we describe a 46 XX patient with NCAH with verumontanum and an absent uterus reared as male. The cited articles were assessed for eligibility.^3^

## 2 Case presentation

**Patient Information.** A 10-year-old phenotypic male patient presented with bilateral impalpable testes and proximal shaft hypospadias.

**Fig. 1:**
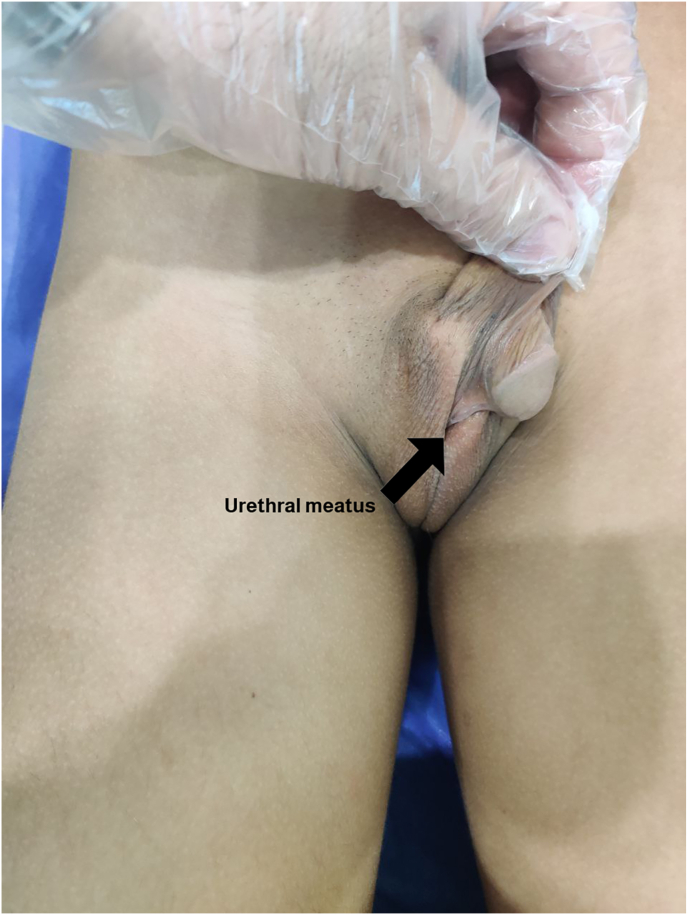
The virilized external genitalia upon inspection.

**Clinical Findings.** Physical examinations revealed an empty scrotum and ambiguous genitalia (Fig. 1). His height was 140cm, and he weighed 36 kg.

**Fig. 2:**
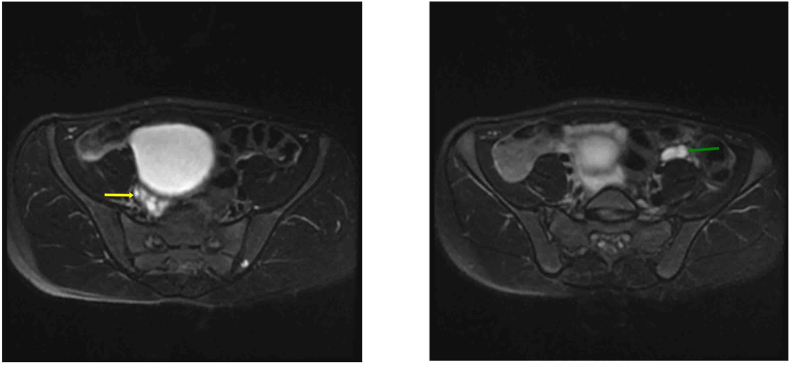
MRI pelvis, fat-suppressed T2WI axial sections in two different levels, show oval shape hyperintense structure on the right side "yellow arrow" and left side "green arrow" of the pelvis, that of right side contains small cystic foci which in favor of follicles inside an ovary.

**Diagnostic Assessment:** Blood tests showed normal complete blood count, electrolytes, renal function, and urine analysis. Ultrasound revealed an empty scrotum, absent uterus, and two intra-abdominal gonads resembling ovaries (Fig. 1). MRI confirmed these findings (Fig. 2). Hormonal tests showed low cortisol (25 nmol/L), elevated ACTH (130 pg/mL), androstenedione (7 ng/mL), testosterone (1.25 ng/mL), and 17OHP (>20 ng/mL). Karyotyping showed 46 XX chromosomes. X-ray indicated advanced bone age.

**Fig. 3:**
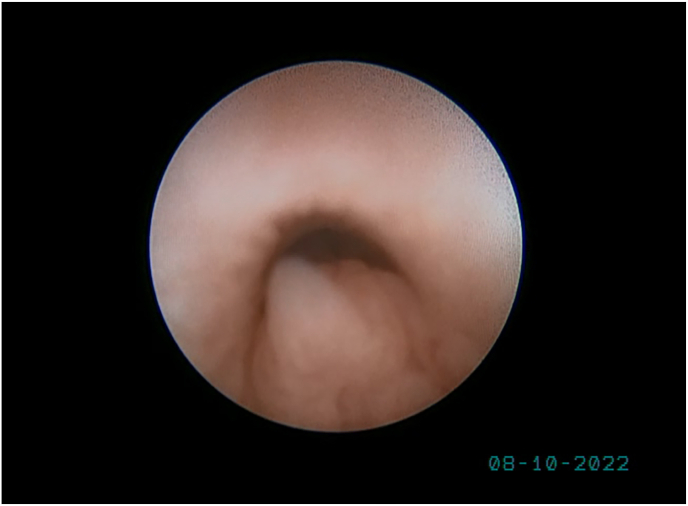
A small round elevation within the urethra located just posterior to the urethral crest (verumontanum).

**Fig. 4:**
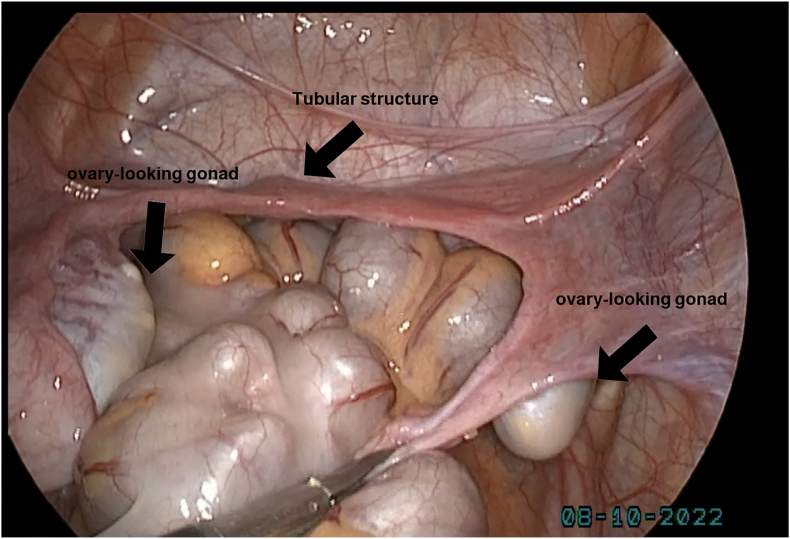
The two gonads with tubular structures between them that were seen on laparoscopy.

**Fig. 5:**
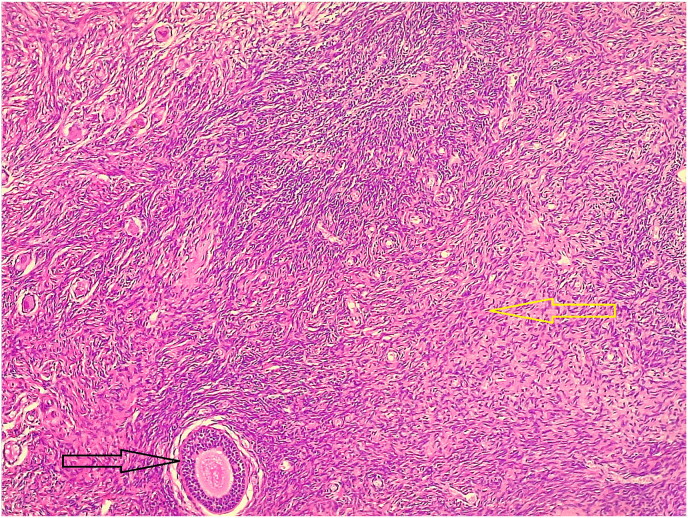
The section shows the primordial follicle (dark arrow) in the ovarian stroma (yellow arrow).

**Diagnostic Intervention:** The case was reviewed by the Multidisciplinary Team (MDT), and cystoscopy and laparoscopy were performed. Cystoscopy showed a normal urethra and verumontanum (Fig. 3). Laparoscopy found two gonads resembling ovaries and no uterus (Fig. 4). Left gonadectomy revealed ovarian tissue, no testicular tissue (Fig. 5). Hydrocortisone treatment (20 mg/day) was started, and follow-up was scheduled. Gender and genital ambiguity management were delayed until the patient can make an informed decision.

## 3 Discussion

The cell cycle's precision parallels the complexity of CAH, an autosomal recessive disorder caused by mutations affecting adrenal steroidogenesis.^2^

Male rearing of 46 XX CAH patients is linked to cultural factors and limited screening. They usually have a uterus, as hysterectomy reports suggest,^2^ but the current patient lacked one. Diagnostic cystoscopy and laparoscopy confirmed the absence of a uterus, making a retrograde contrast study unnecessary, though it may have provided additional insight. Early diagnosis supports female assignment to preserve fertility.^4^

Hypospadias and cryptorchidism complicate 46 XX diagnosis. In this case report, the patient assigned male at birth, lacked screening, delaying CAH detection. While no prostatic tissue was found,^5^ a verumontanum was present—a first reported case in 46 XX CAH without a uterus.

Preventing gender dysphoria is crucial in virilized CAH patients. In the present case, the patient identified as male despite being 46 XX. Most 46 XX CAH patients identify as female, but full virilization can lead to male identity.^4^ Pubertal management is complex; exogenous androgens will be reconsidered as the patient matures, with decisions guided by medical, psychological, and ethical considerations.

Prenatal CAH diagnosis via CYP21A2 analysis is rare in Iraq. Treatment with dexamethasone before 6 weeks prevents masculinization.^6^ While 70 % of treated females have normal genitalia, dexamethasone's impact on fetal neural development is debated.^6^

Sircili et al. recommend feminizing genitoplasty before age 2,^7^ while Sharma et al. advise maintaining male gender after age 5, with male genitoplasty to improve quality of life.^4^ Managament of the patient in this case was delayed to allow gender choice, with unilateral gonadectomy performed to preserve fertility if reassigned female.

## 4 Conclusion

CAH can virilize the external genitalia in 46 XX individuals, sometimes leading to male assignment, and allowing gender choice later may improve psychological outcomes.

## CRediT authorship contribution statement

**Rawa Bapir:** Writing – review & editing, Visualization, Validation, Data curation, Conceptualization. **Ismaeel Aghaways:** Software, Methodology, Investigation, Conceptualization. **Shaho F. Ahmed:** Writing – review & editing, Visualization, Methodology, Formal analysis. **Nali H. Hama:** Writing – review & editing, Visualization, Methodology, Data curation, Conceptualization. **Ari M. Abdullah:** Writing – review & editing, Supervision, Resources, Methodology, Conceptualization. **Soran H. Tahir:** Writing – review & editing, Investigation, Formal analysis, Conceptualization. **Zana B. Najmadden:** Visualization, Resources, Project administration, Methodology, Conceptualization. **Fahmi H. Kakamad:** Writing – review & editing, Writing – original draft, Validation, Software, Conceptualization. **Aso N. Qadir:** Writing – review & editing, Visualization, Validation, Formal analysis, Conceptualization. **Tomas M. Mikael:** Writing – review & editing, Validation, Software, Formal analysis, Conceptualization.
